# Sleep apnea prevalence in chronic kidney disease - association with total body water and symptoms

**DOI:** 10.1186/s12882-017-0544-3

**Published:** 2017-04-04

**Authors:** Hsin-Chia Huang, Giles Walters, Girish Talaulikar, Derek Figurski, Annette Carroll, Mark Hurwitz, Krishna Karpe, Richard Singer

**Affiliations:** 1 Department of Respiratory and Sleep Medicine, The Canberra Hospital, Garran, ACT Australia; 2 Department of Renal Medicine, The Canberra Hospital, Garran, ACT Australia; 3 The Australian National University Medical School, Acton, ACT Australia

**Keywords:** Body water, Chronic kidney failure, Quality of life, Renal dialysis, Sleep

## Abstract

**Background:**

Sleep apnea is common and associated with poor outcome in severe chronic kidney disease, but validated screening tools are not available. Our objectives were to determine the prevalence of sleep apnea in this population, to assess the validity of screening for sleep apnea using an ApneaLink device and to investigate the relationship of sleep apnea to; symptoms, spirometry and body water.

**Methods:**

Patients with glomerular filtration rate ≤30 mL/min/1.73 m^2^, whether or not they were receiving haemodialysis, were eligible for enrolment. Participants completed symptom questionnaires, performed an ApneaLink recording and had total body water measured using bioimpedance. This was followed by a multi-channel polysomnography recording which is the gold-standard diagnostic test for sleep apnea.

**Results:**

Fifty-seven participants were enrolled and had baseline data collected, of whom only 2 did not have sleep apnea. An apnea hypopnea index ≥30/h was found in 66% of haemodialysis and 54% of non-dialysis participants. A central apnea index ≥5/h was present in 11 patients, with only one dialysis patient having predominantly central sleep apnea. ApneaLink underestimated sleep apnea severity, particularly in the non-dialysis group. Neither total body water corrected for body size, spirometry, subjective sleepiness nor overall symptom scores were associated with sleep apnea severity.

**Conclusions:**

This study demonstrates a very high prevalence of severe sleep apnea in patients with chronic kidney disease. Sleep apnea severity was not associated with quality of life or sleepiness scores and was unrelated to total body water corrected for body size. Routine identification of sleep apnea with polysomnography rather than screening is more appropriate in this group due to the high prevalence.

**Electronic supplementary material:**

The online version of this article (doi:10.1186/s12882-017-0544-3) contains supplementary material, which is available to authorized users.

## Background

Sleep apnea is defined as an apnea-hypopnea index (AHI) ≥5 episodes per hour in symptomatic patients or ≥15 obstructive episodes per hour in asymptomatic patients [1, 2]. Sleep apnea severity is determined by the degree of sleepiness and by the AHI [1]. Determining whether symptoms of sleep apnoea are present is problematic in patients with severe kidney failure since some accepted symptoms such as unrefreshing, restless sleep and fatigue are often reported by patients with severe chronic kidney disease (CKD), even in the absence of significant sleep apnea [3, 4]. Other symptoms such as nocturia [2] cannot be present in anuric dialysis patients or may be present but unrelated to sleep apnoea in severe, non dialysis kidney failure. These issues make symptom based screening questionnaires less discriminatory in a CKD population [5] and for this reason a sleep apnea definition of AHI ≥ 5, irrespective of symptoms is used in this study.

The prevalence of apparently asymptomatic sleep disordered breathing in non-CKD women and men is increasing in prevalence and is thought to be approximately 26% of the US adult population [6, 7]. Sleep apnea is a risk factor for the development of CKD, progression of CKD [8] and is associated with cognitive impairment in those with CKD [9]. Treatment in the non-CKD population is associated with lower blood pressure [10] and perhaps with improved survival, [11] but the effect in those with CKD is unknown. A high prevalence of sleep apnea has been recognized in patients with CKD since the 1980s with the prevalence of severe sleep apnea (AHI ≥ 30) reported to be present in 20% of those with severe non-dialysis CKD and 26% of those on hemodialysis [12]. Other studies, of varying quality, have reported a prevalence for AHI ≥ 5 in those on hemodialysis of between 50 and 83% [13–16] and there is data to suggest that a lower estimated glomerular filtration rate (eGFR) is associated with more severe sleep apnea in CKD patients [17].

Severe muscle weakness and fluid retention have been proposed as explanations for the high prevalence of sleep apnea in CKD patients. Airway muscle weakness makes airways more prone to collapse on inspiration, whereas fluid retention appears to result in swelling of airway walls and therefore increases the resistance to airflow and propensity to collapse [18]. In accordance with this, obstructive lung disease in non-dialysis patients [19] and fluid overload in CKD patients [20, 21] have been associated with more severe nocturnal hypoxaemia.

The gold standard for diagnosis of sleep apnea is an attended overnight polysomnogram (PSG), [22] but this is a labour intensive and costly investigation. Limited channel recordings such as ApneaLink (ResMed Corp, Poway, California), provide some information on respiratory parameters, but no information on duration of sleep. These devices are available to screen for the presence of sleep apnea in some settings, [23] but have not been validated in a CKD population. Availability of a validated screening device may enable a cost-effective screening programme for sleep apnea to be developed in the CKD population.

The objectives of this study were to determine the prevalence of sleep apnea in a severe CKD population, to assess the validity of screening for sleep apnea using an ApneaLink device and to investigate the relationship of sleep apnea to; symptoms, spirometry and body water.

## Methods

The Canberra Hospital is a publicly funded 600 bed tertiary hospital servicing a population of approximately 570000. Patients were approached for enrolment if they were; ambulant, aged over 18 years, under the care of the Canberra Hospital Renal Service, and had an eGFR by 3 variable MDRD formula [24] of <30 mL/min/1.73 m^2^. For those receiving haemodialysis, they must have been established on haemodialysis for at least 3 months. Participants were excluded if they; had been hospitalized within the previous month, were unable or unwilling to provide informed consent, had previously diagnosed sleep apnea, usually received greater than 18 h haemodialysis per week or were treated with peritoneal dialysis. Enrolment was planned to continue until at least 50 subjects had undergone a PSG, split equally between the haemodialysis and non-dialysis groups. This number was based on available funding and investigator expectations of dialysis patient consent rates. Patients that declined to participate were asked during the consent process whether they rated their sleep as “good” or “poor”. As recommended in guidelines for this type of study, patients formed a consecutive series and were not selected or approached based on the presence or absence of symptoms, co-morbidities, sex or age [25].

At the initial study visit data on; height, neck circumference most recent post-dialysis weight (or current weight for non-dialysis subjects), eGFR and smoking history were collected. Data on subject race was not collected as previous studies in patients with severe renal failure at the same institution demonstrated that subjects were often either unwilling to answer questions regarding ethnic/racial origin or gave ambiguous answers that required categorisation by the investigators. For this reason, as is standard for automated eGFR reporting in Australia, race was set to “white”. Body mass index (BMI) was calculated as *Weight* (*kg*)/*Height* (*m*) ^*2*^. Participants were also given; the Kidney Dialysis Quality of Life-Short Form [26] (KDQOL-SF), Epworth Sleepiness Scale [27] and a modified Berlin Questionnaire [28] to complete.

The Berlin Questionnaire asks about the presence of snoring, sleepiness and hypertension. A positive score in 2 out of the 3 symptom domains denotes a high risk for sleep apnea. Since hypertension and/or use of antihypertensives were universal in the study group and thus non-discriminatory, we modified the Berlin Questionnaire to exclude hypertension from the scoring. The Epworth Sleepiness Scale assesses the likelihood of falling asleep during common activities. Persons without sleep disorders have a mean score of approximately 6 out of 24 [27].

Participants were shown how to use the ApneaLink device, and took it home for a self-administered overnight recording within 6 weeks of collection of baseline data. The ApneaLink is a Type 4 limited channel recording device [29] that measures nasal airflow and oximetry only. Output was auto-analysed using software provided by the manufacturer.

To avoid delaying treatment of participants with potentially severe sleep apnea, those that were “High Risk” on the Berlin Questionnaire, or who had an ApneaLink AHI ≥10, or who had an Epworth score ≥10 and an ApneaLink AHI ≥5 were seen by a sleep physician within 1 week. An overnight PSG was performed in all participants 1 week after the ApneaLink. Both the ApneaLink and PSG were performed on the same day of the week and in all cases this was on an evening immediately following a dialysis session (for hemodialysis participants). Haemodialysis participants attended an in-lab PSG (E-Series, Compumedics, Melbourne, Australia), whereas an unattended PSG (SomtePSG, Compumedics, Melbourne, Australia) was performed in non-dialysis participants. Investigators were not blinded to the ApneaLink and clinical data. The sleep scoring was performed using the Compumedics ProFusion 3 software in accordance with AASM criteria [30]. Central sleep apnea index (CAI) was defined as the number of central apneas per hour of sleep.

Spirometry and body water was measured on the day of the sleep study by a trained technician (post dialysis for haemodialysis participants). Spirometry was performed and reported according to ATS/ERS guidelines [31]. Body water was estimated using bioimpedance (BCM, Fresenius Health Care, Bad Homburg, Germany) [32]. To reduce the dependence of total body water on body size, total body water was divided by body weight (referred to as body water fraction) or height squared. The post-dialysis weight was used in haemodialysis participants. As female dialysis patients have a lower body water fraction than males, [33] analysis was performed separately in males and females. Spirometry was measured using the EasyOne (Medizintechnik, Switzerland) spirometer with predictive normal values based on the 3rd National Health and Nutrition Examination Survey [34].

For the purposes of assessing selection bias in participant recruitment, the study population was considered to be haemodialysis and severe non-dialysis CKD patients serviced by The Canberra Hospital Renal Service as at 31st December, 2013.

### Statistics

Normally distributed data is presented as mean ± standard deviation and non-normally distributed data as median (interquartile range). Raw or transformed data distribution was tested using the Shapiro-Wilk and Levene’s tests. Agreement was tested using Cohen’s kappa. Differences in means and medians were tested by *t*-test, ANOVA, Wilcoxon Rank-Sum or Kruskal-Wallis Rank test as appropriate. Nominal variables were tested by Fisher Exact test. Statistical significance was set at a *p* value of <0.05, with all *p* values 2-sided except when inappropriate (in >2 way contingency tables). Significance level was not adjusted for multiple testing.

## Results

**Fig. 1:**
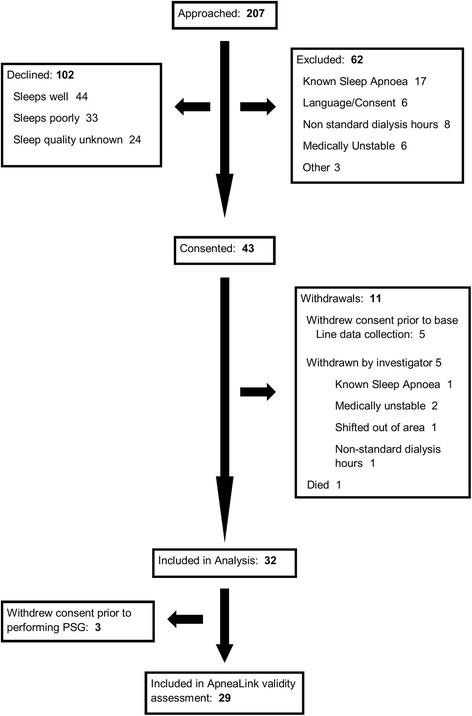
Study flow in dialysis participants

**Fig. 2:**
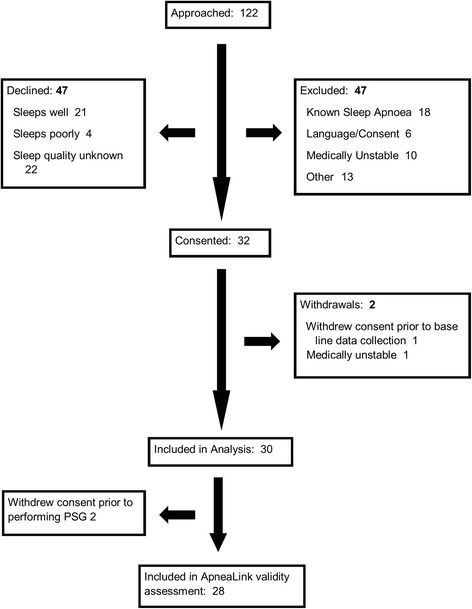
Study flow in non-dialysis participants

Participant flow through screening, enrolment and consent procedures are shown in Figs. 1 and 2. A total of 32 non-dialysis patients consented to enrol during the non-dialysis recruitment period (September 2012 to November 2013). Two participants left the study prior to collection of baseline data and were not included in data analysis. Another 2 participants withdrew following partial collection of baseline data, but before performing polysomnography. These participants were included in the analyses for collected data.

A total of 43 haemodialysis patients consented to enrol during the haemodialysis recruitment period (May 2010 to January 2012). Eleven participants left the study prior to collection of baseline data and were not included in data analysis. Another 3 participants withdrew following partial baseline data collection, but before performing the polysomnography. These participants are included in the analyses for collected data.

Participant baseline characteristics are summarised in Table 1. The majority were not obese, but were relatively sleepy, with close to 30% in each group having Epworth scores of ≥10. A similar proportion of dialysis and non-dialysis participants were categorized as “high risk” for sleep apnea by the modified Berlin Questionnaire.

**Table 1:** Baseline Characteristics

	Dialysis Group (*n* = *32*)	Non-Dialysis Group (*n* = 30)	*P* value
Age (years)	65.5 (50 to 71.5)	71 (55 to 78)	0.11
Male/Female	29/3	22/7	0.12
BMI (kg/m^2^)	26.8 (24.1 to 30.9)	29.5 (25.2 to 32.6)	0.13
Neck Circumference (cm)	41 (38 to 45.5)	39.5 (38 to 42)	0.05
eGFRmL/min/1.73 m^2^		21.2 ± 5.6	
Dialysis dose (Kt/V)	1.32 ± 0.18		
Dialysis Vintage (years)	1.8 (1.1 to 3.7)		
Epworth Sleepiness Scale	8 (5 to 10)	7 (6 to 10)	0.79
Epworth Sleepiness Scale ≥10	10 (31%)	8 (27%)	0.69
High Risk Category on modified Berlin Questionnaire	34%	40%	0.79

Males were significantly over-represented in the study sample, but median age and BMI were similar to those seen in the study population (Table 2).

**Table 2:** Study Sample Compared to Study Population

	Haemodialysis Population (*n* = 266)	Haemodialysis Study Sample (*n* = 32)	*p* value	Non-dialysis Population (*n* = 435)	Non-Dialysis Study Sample (*n* = 30)	*p* value
Age	66 (55 to 76)	65.5 (50 to 71.5)	0.33	72 (62 to 80)	71 (55 to 78)	0.42
BMI	26.3 (22.3 to 30.6)	26.8 (24.1 to 30.9)	0.36	28.3 (24.9 to 32.2)	29.5 (25.2 to 32.6)	0.46
% Male	64%	91%	0.002	55%	73%	0.003

Ninety-six percent of participants had some degree of sleep disordered breathing and this was predominantly obstructive. Significant central sleep apnea - a central apnea index ≥5/h by PSG- was present in 11 patients, most of whom were in the dialysis group. Only one dialysis patient had predominantly central sleep apnea, with CAI of 88/h. Most participants were severely hypoxic at some point during sleep. The median nadir oxygen saturation was 80% and the median percentage of sleep time spent below 90% oxygen saturation was 4% (IQR 7 to 19%). The median periodic leg movements index (PLMI) was low in both groups (2/h) but 25% of patients still had a PLMI of ≥15/h. Sleep onset latency and sleep efficiency was similar in both groups. The ApneaLink and PSG derived data is summarised in Table 3.

**Table 3:** ApneaLink and PSG Sleep data

	Dialysis Group	Non-Dialysis Group	Overall	*p* value (Dialysis vs Non Dialysis)
ApneaLink AHI	22.5 (12 to 41.5)	12.5 (4 to 33)	19.5 (7 to 38)	0.028
ApneaLink AHI ≥ 15	21 (66%)	14 (47%)	35 (56%)	0.2
PSG AHI	37.6 (23.1 to 77.2)	34.4 (19.6 to 58.7)	35.3 (20.5 to 69.1)	0.24
PSG AHI <5	1 (3%)	1 (4%)	2 (4%)	0.62 (one tailed)
PSG AHI 5 to <15	5 (17%)	4 (14%)	9 (16%)	
PSG-AHI 15 to <30	4 (14%)	8 (29%)	12 (21%)	
PSG-AHI ≥30	19 (66%)	15 (54%)	34 (60%)	
PSG AHI ≥15	23 (79%)	23 (82%)	46 (81%)	1
PSG CAI	0.7 (0.4 to 5.4)	0.35 (0 to 1.5)	0.6 (0.2 to 2.3)	0.04
PSG CAI ≥ 5	9 (31%)	2 (7%)	11 (19%)	0.02

**Fig. 3:**
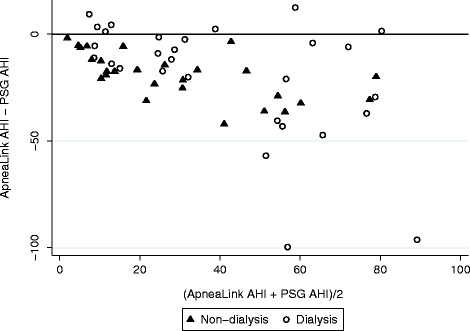
Bland-Altman plot of ApneaLink and PSG AHI

The sensitivity and specificity of ApneaLink for categorizing participants as having an AHI ≥ 15 is summarised in Table 4. Agreement between ApneaLink and PSG determined sleep apnea severity category was moderate in the dialysis group, (kappa = 0.39, *p* = 0.0004) but low and not statistically different from chance in the non-dialysis group, (kappa = 0.1, *p* = 0.15). Agreement improved for identifying moderate and severe sleep apnea (AHI ≥15), with kappa of 0.32, (*p* = 0.01) in the non-dialysis group and kappa of 0.63 (*p* = 0.0003) in the dialysis group. ApneaLink underestimated the AHI in all but 7 participants, all of whom were in the dialysis group (Fig. 3).

**Table 4:** Test characteristic of ApneaLink for AHI ≥ 15

	Sensitivity	Specificity	Positive Predictive Value	Negative Predictive Value
Overall	72%	91%	97%	43%
Dialysis group	87%	83%	95%	63%
Non-dialysis group	57%	100%	100%	33%

Higher BMI and neck circumference correlated with a higher AHI in non-dialysis participants, but not in those on dialysis (Table 5). Age was not statistically associated with sleep apnea severity in either group.

**Table 5:** Correlation of BMI and neck circumference to AHI

	Dialysis Group (*n* = 28)		Non Dialysis Group (*n* = 29)	
	Spearman rho	*p* value	Spearman rho	*p* value
BMI	0.2	0.29	0.48	0.01
Neck Circumference	0.36	0.06	0.67	0.0001

Spirometry data, expressed as a percentage of predicted values based on age, sex and height, is summarized in Table 6. Dialysis participants had more restrictive spirometry, particularly in the 5 patients with AHI < 15.

**Table 6:** Association of Spirometry Data with PSG AHI

	Dialysis Group (*n* = 28)			Non-Dialysis Group (*n* = 24)		
	AHI < 15 (*n* = 5)	AHI > =15 (*n* = 23)	*p* value	AHI < 15 (*n* = 5)	AHI > =15 (*n* = 18)	*p* value
FEV1 (% predicted)	59 ± 12	75 ± 16	0.047	79 ± 22	82 ± 18	0.78
FVC (% predicted)	59 ± 5	73 ± 15	0.07	84 ± 13	77 ± 15	0.32
FEV1/FVC	75 ± 12	79 ± 5	0.30	72 ± 11	78 ± 9	0.16

Non-dialysis participants with AHI ≥ 15 recorded improved quality of social interaction (score of 86 compared to 66, *p* = 0.03) and a trend towards improved sleep (score of 62 compared to 47, *p* = 0.051). Aside from this, sleep apnea severity was not associated changes in any domain of the KDQOL-SF. Neither the modified Berlin Questionnaire, nor the Epworth Sleepiness Scale discriminated between those with less, or more severe sleep disordered breathing.

There was no difference in the body water fraction between male dialysis participants and non-dialysis participants, and no association to sleep apnea severity or to the presence of central sleep apnea (Table 7). Analysis of female participants was not performed due to the very small sample size. Using height, or height squared, rather than total body weight, to correct for body size did not alter these findings. Similarly, no relationship was seen between fat mass or extra-cellular fluid mass as a fraction of body weight and sleep apnea severity. Data for the single participant with severe central sleep apnea (CAI = 88) indicated that his body water fraction was similar to the group median.

**Table 7:** Body Water Fraction and Severity of Sleep Apnea in Males

	Overall (*n* = 39)			Dialysis group (*n* = 15)			Non-dialysis group (*n* = 24)		
AHI	<15	≥15	*p*	<15	≥15	*p*	<15	≥15	*p*
Body water fraction (L/Kg)	0.50 (0.49 to 0.54)	0.46 (0.44 to 0.51)	0.22	0.54 (0.42 to 0.55)	0.47 (0.43 to 0.49)	0.32	0.50 (0.49 to 0.50)	0.46 (0.45 to 0.52)	0.87
Body Water/Height^2^ (L/m^2^)	12.9 (11.8 to 15.8)	13.0 (11.9 to 14.2)	0.97	11.8 (9.4 to 17.5)	12.5 (10.9 to 13.2)	0.76	14.3 (12.9 to 15.8)	14 (13.4 to 15.4)	0.87

## Discussion

We found a very high, 96%, prevalence of sleep disordered breathing and 80% of participants had moderate or severe sleep apnea (AHI ≥ 15). These prevalence figures are higher than most previous reports [12, 14–16] Central sleep apnea was much more common in haemodialysis compared to non-dialysis participants (31% versus 7%), but CAI ≥ 30 was present in only a single patient. The reported prevalence for central sleep apnea in subjects with CKD was 0 to 75% in a recent systematic review [35]. However, this range is unhelpful in drawing firm conclusions on prevalence since the definition of sleep apnea varied across studies included in the review and the study with 75% prevalence was based on only 8 subjects [35].

Our overall enrolment rate was lower than several other publications, which reported recruitment rates of 50-67% [5, 16, 21, 36]. We enrolled only 40% of patients approached in the non-dialysis group and 22% in the haemodialysis group. The non-dialysis enrolment rate is, however, similar to that seen in the large Wisconsin Sleep Cohort study [6]. We attempted to obtain a representative sample from the study population but were unsuccessful, particularly in the haemodialysis group, where a substantial excess of men enrolled. Previous studies in dialysis patients have also enrolled an excess of men, [5, 16, 36] but it is uncertain if this was due to selection bias since the composition of their study populations were not reported. The male enrolment proportion in those studies (approximately 70%) is substantially lower than the 91% seen in our study. Despite our study sample mirroring the research population for the other demographic variables collected, it is likely that the prevalence data is biased, since sleep apnea is thought to be much more common in men [6].

It is unclear why women were difficult to enrol. Perhaps potential male participants felt they were likely to have sleep apnea due to symptoms that were not captured in our questionnaires and therefore they had a greater incentive to participate. The minimum estimated sleep apnea prevalence in the study population was 28% (non-dialysis) and 48% (dialysis) when those known to have sleep apnea were included. In this calculation non-consenting potential subjects were assumed not to have sleep apnea and were included in the denominator.

Traditional risk factors of higher BMI and neck circumference were associated with more severe sleep apnea in non-dialysis participants but not in dialysis participants. This suggests a different mechanism promoting sleep apnea may be present in those on dialysis, compared to the CKD or normal population.

It has been reported that pharyngeal caliber responds to diuretic induced fluid removal during the treatment of heart failure and that sleep apnea severity improves with fluid removal in newly initiated peritoneal dialysis patients [20, 37]. In newly initiated peritoneal dialysis patients pharyngeal edema is associated with more severe sleep apnea but a relationship to total body water in that study was not reported [20]. The subjects in that study were of lower body weight and higher body water fraction (approximately 62%) than subjects in our study. In contrast, we found that bioimpedance determined total body water as a proportion of body size was not predictive of sleep apnea severity. It is likely that total body bioimpedance measurements are either not sufficiently sensitive to detect changes in the pharyngeal edema or alternatively, that total body water is only a minor determinant of sleep apnea severity in patients with severe kidney failure. It is a limitation of our study that pharyngeal fluid was not directly assessed, and hence the relationship to total body water could not be confirmed.

The ApneaLink AHI was lower than PSG derived AHI in all but 7 participants, most likely because the screening device cannot distinguish sleep from wakefulness. It was not possible to validate the ApneaLink device as a screening tool for the presence of sleep apnea because so few participants had a normal AHI. However, unlike symptom questionnaires (which other investigators have also noted to be inaccurate in CKD [5]) the device had good agreement with PSG in detecting participants with AHI ≥ 15. Agreement was, however, only fair in non-dialysis participants and ApneaLink appeared unsatisfactory as a screening tool for this group. The specificity and negative predictive values reported in Table 4 should be treated with caution due to the high dependency on results in only a few patients. The relatively low negative predictive values in a population with very high prevalence indicates that, despite the good agreement seen in haemodialysis participants, ApneaLink screening is unlikely to be useful, and patients should proceed directly to polysomnography.

We did not find evidence of worse scores in the symptom or other KDQOL-SF domains in participants who had more severe sleep apnea. Participants were very symptomatic in terms of the average Epworth Sleepiness Scale scores but there was no evidence of greater sleepiness or impaired subjective cognition in those with higher AHIs. In fact, the apparent direction of some statistically significant associations was towards improved quality of life in those with more severe sleep apnea. These statistical associations are probably due to chance, especially since we applied multiple statistical tests in this study. There is no plausible explanation why more severe sleep apnea should improve any aspect of quality of life. Our results suggest that neither the KDQOL-SF nor the Epworth Sleepiness Scale is sensitive to changes AHI. Similar results with respect to the Epworth Sleepiness Scale have been reported in a similar CKD population [12]. We did not screen for restless leg syndrome, but a quarter of participants had ≥15 leg movements/h) – which is a somewhat lower rate than reported in a previous study in dialysis patients [16].

## Conclusions

Systematic identification of sleep apnea in CKD patients should be considered given that it is associated with a poor outcome [8, 9] however we were unable to validate the ApneaLink device as a screening tool in this population due to the very high prevalence of sleep disordered breathing. The lack of an association between sleep apnea severity and symptom questionnaires does not mean that participants were asymptomatic. A more plausible explanation is that these instruments are insensitive to the effects sleep apnea in patients with severe CKD. Therefore, future intervention studies performed in this population will likely need to utilize other instruments, or alternative end points such as progression of renal disease to assess the effectiveness of therapy. Our results suggest that measures of total body water are not associated with sleep apnea severity in this population.

## Additional files

Additional file 1: Study sample data. (XLSX 51 kb)

Additional file 2: Comparison data of study population to the study sample. (XLSX 26 kb)

## Abbreviations

AHI: Apnea hypopnea index
CAI: Central apnea index
CKD: Chronic kidney disease
KDQOL-SF: Kidney disease quality of life instrument– short form
PSG: Poly somnogram
